# A Systematic Survey of Mini-Proteins in Bacteria and Archaea

**DOI:** 10.1371/journal.pone.0004027

**Published:** 2008-12-24

**Authors:** Fengyu Wang, Jingfa Xiao, Linlin Pan, Ming Yang, Guoqiang Zhang, Shouguang Jin, Jun Yu

**Affiliations:** 1 CAS Key Laboratory of Genome Sciences and Information, Beijing Institute of Genomics, Chinese Academy of Sciences, Beijing, China; 2 Graduate School of Chinese Academy of Sciences, Beijing, China; 3 James D. Watson Institute of Genome Sciences of Zhejiang University, Hangzhou, China; 4 Department of Molecular Genetics and Microbiology, University of Florida College of Medicine, Gainesville, Florida, United States of America; University of Vermont, United States of America

## Abstract

**Background:**

Mini-proteins, defined as polypeptides containing no more than 100 amino acids, are ubiquitous in prokaryotes and eukaryotes. They play significant roles in various biological processes, and their regulatory functions gradually attract the attentions of scientists. However, the functions of the majority of mini-proteins are still largely unknown due to the constraints of experimental methods and bioinformatic analysis.

**Methodology/Principal Findings:**

In this article, we extracted a total of 180,879 mini-proteins from the annotations of 532 sequenced genomes, including 491 strains of Bacteria and 41 strains of Archaea. The average proportion of mini-proteins among all genomic proteins is approximately 10.99%, but different strains exhibit remarkable fluctuations. These mini-proteins display two notable characteristics. First, the majority are species-specific proteins with an average proportion of 58.79% among six representative phyla. Second, an even larger proportion (70.03% among all strains) is hypothetical proteins. However, a fraction of highly conserved hypothetical proteins potentially play crucial roles in organisms. Among mini-proteins with known functions, it seems that regulatory and metabolic proteins are more abundant than essential structural proteins. Furthermore, domains in mini-proteins seem to have greater distributions in Bacteria than Eukarya. Analysis of the evolutionary progression of these domains reveals that they have diverged to new patterns from a single ancestor.

**Conclusions/Significance:**

Mini-proteins are ubiquitous in bacterial and archaeal species and play significant roles in various functions. The number of mini-proteins in each genome displays remarkable fluctuation, likely resulting from the differential selective pressures that reflect the respective life-styles of the organisms. The answers to many questions surrounding mini-proteins remain elusive and need to be resolved experimentally.

## Introduction

Mini-proteins are polypeptides consisting of no more than 100 amino acids (AA), which are widespread in both prokaryotes and eukaryotes and found to play important roles in a variety of functionalities. Mini-proteins usually contain a single domain. In prokaryotes, well known mini-proteins include chaperonin Hsp10, translation initiation factor IF-1, ribosomal proteins and others. In eukaryotes, certain important signalling molecules, animal toxins and protease inhibitors belong to the mini-protein family [1]. James Kastenmayer reported that the *Saccharomyces cerevisiae* genome codes for 299 mini-proteins based on experimental approaches and computational analysis [2].

Some mini-proteins have been used as model systems to study the determinants of protein folding and stability because of their simple and typical structures [3], [4]. Moreover, some exhibit structural scaffolds valuable to the study of binding activities, identification of frameworks for peptidomimetic design, or search for novel drug candidates [5]. Besides their importance in structural studies, reports on the regulatory functions of mini-proteins have recently aroused extensive interests, especially in Bacteria. For instance, Wu et al. [6], [7] have elucidated the functions of two mini-proteins from *Pseudomonas aeruginosa*. These proteins were expressed in response to specific environmental stresses and actively participate in the suppression of the type III secretion system, achieving coordinated gene expression, thus playing a critical role in host infection. Within dormant spores of *Bacillus*, *Clostridium* and related species, a group of small, acid-soluble spore proteins (SASP) are the crucial factors enabling spores to survive for years, protecting spore DNA from damaging agents [8].

According to binding studies of peptides of various sizes, the minimal size of a functional epitope is around 8 AA, with an average size of 15–20 AA. Therefore, a mini-protein as short as 8 AA is capable to binding targets and to exhibit biological functions. It is not surprising then that mini-proteins with sizes up to 100 AA can perform a variety of relevant functions and participate in regulation of various biological processes. However, little effort had been put to explore their functions; instead, most researches focus on large proteins that are conserved and/or essential among organisms [9]. The characterization of mini-proteins presents difficulties in experimental and bioinformatic approaches. Experimentally, mini-proteins are difficult to isolate and identify due to their small sizes; likewise, in bioinformatic analyses, short genes are the most difficult to predict. Therefore, to provide a clue for their functions, it is necessary to conduct in depth and systematic studies of the mini-proteins.

In this report, we analyzed all annotated protein sequences that are ≤100 amino acids (AA) from 532 completed genome data, including 491 sequences of Bacteria and 41 sequences of Archaea, deposited in the Microbial Genome Database at the National Center for Biotechnology Information (NCBI) [10]. We focused our attention on three aspects: the component distribution of mini-proteins (including length, number, and conservation), the characteristics of mini-proteins in bacterial and archaeal species, and the possible reasons why they possess such characteristics. The results indicate that mini-proteins account for an average of 10.99% of all annotated sequences in Bacteria and Archaea, comprising numerous species-specific proteins and hypothetical proteins. The functions of very few mini-proteins are known, but these involve many important biological processes. Moreover, hypothetical mini-proteins contain a fraction of highly conserved sequences, indicating that they play important functional roles.

## Results

### Mini-protein length distribution

**Figure 1:**
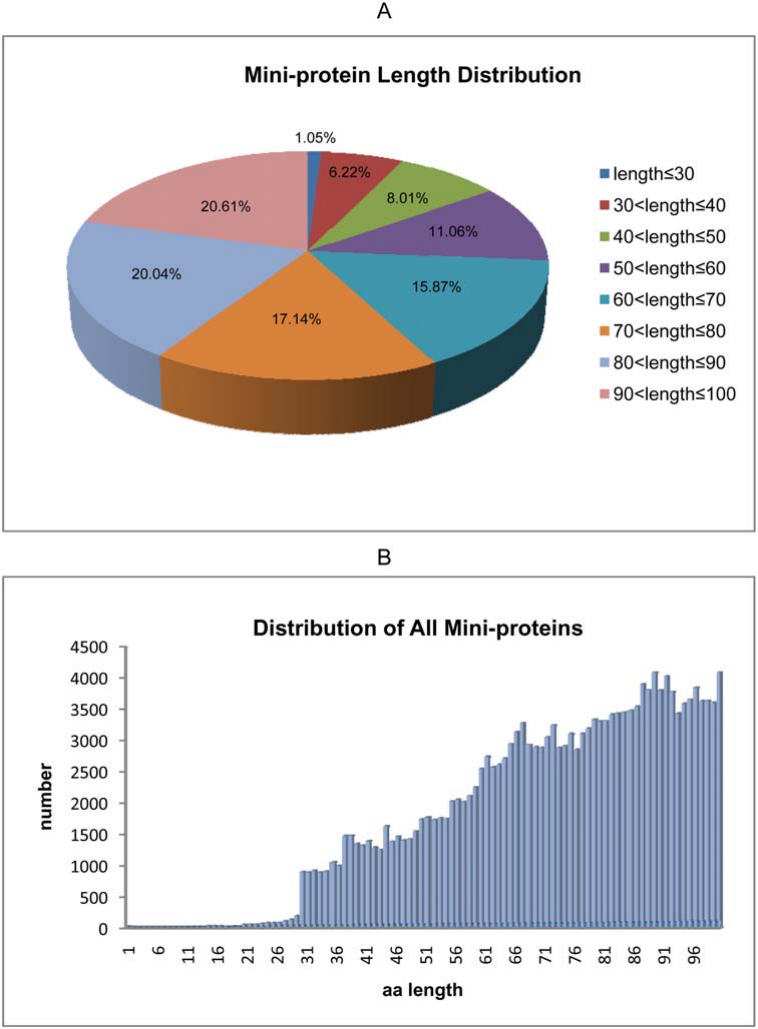
A: Mini-protein length distribution. B: Distribution of all mini-proteins.

We downloaded 532 sequenced genome data of prokaryotes, consisting of 491 strains of Bacteria and 41 strains of Archaea, from National Centre for Biotechnology Information (NCBI). A total of 180,879 annotated protein sequences with no more than 100 amino acids were extracted. The length distribution of these mini-proteins shows increase in frequency for progressively longer sequences (Figure 1A). Mini-proteins with ≤30AA are the minority in all data, representing merely 1,897 sequences, and accounting for 1.05% of all mini-proteins. The longest sequences, 90AA<length≤100AA, are more common than other categories, with 37,280 sequences accounting for 20.61% of all mini-proteins. Figure 1B displays the detailed length distribution of mini-protein, with lengths from 1AA to 100AA. The general trend that mini-protein numbers increase with mini-protein length is obvious. Only 5 mini-protein sequences were ≤10AA with the shortest protein containing 6 amino acids. Three of these are hypothetical proteins. Of the other two mini-proteins, one is predicted to be a fragment of the PE-PGRS protein family whose members are probably related to surface antigens in mycobacterial species; the other is annotated as transposase-like protein B (remnant) in *Clostridium difficile 630*. Proteins of 100 amino acids are the most abundant, with 4,092 sequences.

### Mini-protein overview in phylum

The 532 sequenced genomes we collected from NCBI belonged to species classified in 18 distinct phyla, 3 from Archaea and 15 from Bacteria. Four phyla were represented by single genome sequences, i.e., Nanoarchaeota from Archaea and Aquificae, Fusobacteria and Planctomycetes from Bacteria. Moreover, we treated Proteobacteria's five classes as phyla to describe, namely Alpha-, Beta-, Delta-, Epsilon- and Gamma-, because they are represented by the largest number of genomes, with 258 strains accounting for nearly half of sequenced genomes.

As shown in Table 1, the overall proportion of mini-proteins among all annotated genomic proteins is 10.99%. Planctomycetes has the highest number of mini-proteins, comprising 26.54% or 1,944 sequences. In contrast, Aquificae has the least number of mini-proteins, merely encoding 48 mini-proteins in the whole genome, representing 3.08%. However, these two phyla contain only one genome each, *Rhodopirellula baltica* and *Aquifex aeolicus*, respectively. Except for these two extremes, other phyla encode similar proportions of mini-proteins, although greater variability is observed when considering individual strains. For instance, the Alphaproteobacterium *Anaplasma phagocytophilum* contains 33.39% mini-proteins, more than any other genome. On the other extreme, in the genome of *Clostridium tetani* (Firmicutes), a human pathogen causing tetanus, there are no mini-proteins annotated except for 4 sequences on its plasmid. Genomes from nine other species of *Clostridium* have been sequenced. In sharp contrast to the *C. tetani*, these nine genomes contain a normal proportion of mini-proteins, ranging from 14.25% to 8.27%. Moreover, similar average proportions of mini-proteins, 11.28%, 11.30% and 9.33%, respectively, are annotated in the genomes of three archaeal phyla.

**Table 1:** Overview of mini-proteins in phylum.

Domain	Phylum	Sum	Average%	Range%	Average Length	Minimum Length	Organism Number	Average Sum
Archaea	Crenarchaeota	2953	11.28	8.36–18.23	77	18	12	246
Archaea	Euryarchaeota	7642	11.30	7.83–15.00	76	16	28	273
Archaea	Nanoarchaeota	50	9.33	9.33	81	54	1	50
Bacteria	Acidobacteria	696	5.58	5.35–5.80	84	37	2	348
Bacteria	Actinobacteria	14195	8.01	4.53–15.88	76	10	42	338
Bacteria	Aquificae	48	3.08	3.08	80	47	1	48
Bacteria	Bacteroidetes	3582	8.89	5.51–13.11	74	27	11	326
Bacteria	Chlamydiae	1296	9.98	6.22–17.73	73	30	11	118
Bacteria	Chlorobi	1295	11.79	6.79–21.80	72	30	5	259
Bacteria	Chloroflexi	979	13.29	6.31–18.16	74	27	4	245
Bacteria	Cyanobacteria	12057	17.31	7.80–30.83	71	15	26	464
Bacteria	Firmicutes	36465	12.60	0.16–25.11	70	6	113	323
Bacteria	Fusobacteria	232	11.22	11.22	72	20	1	232
Bacteria	Planctomycetes	1944	26.54	26.54	66	35	1	1944
Bacteria	Alphaproteobacteria	21246	11.03	5.09–33.39	74	20	65	327
Bacteria	Betaproteobacteria	21347	10.02	5.02–24.49	73	13	44	485
Bacteria	Deltaproteobacteria	5664	10.12	1.80–18.99	72	18	15	378
Bacteria	Epsilonproteobacteria	2099	11.03	7.35–16.75	69	12	11	191
Bacteria	Gammaproteobacteria	42626	9.75	4.58–26.91	73	9	123	347
Bacteria	Spirochaetes	3225	13.63	5.49–28.71	61	14	9	358
Bacteria	Thermi	761	7.46	5.75–9.29	78	11	4	190
Bacteria	Thermotogae	477	8.62	7.28–9.28	73	30	3	159
		180879	10.99%		74		532	346

### Specific and shared mini-proteins

To investigate conservation among mini-proteins, we took several representative phyla to determine the proportion of their mini-proteins that are specific or shared to each taxonomic level (species, genus, family, order, class, phylum and domain). Conservation was established by sequence similarity as determined by BLAST comparisons (see Table 2). Our criteria for the definition of specific vs. shared include the following: (i) Except for species-specific proteins, the specificity at other taxonomic levels must meet two conditions, namely not only are they particular at a certain level, but they also simultaneously exist in all categories at the lower levels. For instance, as a query sequence, one mini-protein belongs to a certain species and a certain genus, and the results indicate that its homologs are only present in all species in the same genus. In this case, we call it a “genus-specific” protein. Similarly, if its homologs are found in other genera in the same family, then we name it the “genus-shared”; (ii) Given that a genus might only have one sequenced species, a mini-protein named “species-specific” does not automatically become genus-specific. This rule also applies to other levels; (iii) Because of filtration by various parameters, the entries shown in the results are less than the number of mini-proteins used in the initial searches.

**Table 2:** Specific or shared mini-proteins in phyla.

Mini-Proteins Categories	Euryarchaeota	%	Actinobacteria	%	Cyanobacteria	%	Firmicutes	%	Gamma Proteobacteria	%	Spirochaetes	%
Sum	7643		14195		12057		36465		42626		3225	
Blast-result	7638		14184		12055		36383		42534		3212	
Species-specific	4854	63.55	7551	53.24	7245	60.10	20304	55.81	18711	43.99	2443	76.06
Species-shared	238	3.12	2619	18.46	907	7.52	5930	16.30	4684	11.01	69	2.15
Genus-specific	431	5.64	661	4.66	–	–	2354	6.47	1965	4.62	524	16.31
Genus-shared	413	5.41	–	–	23	0.19	945	2.60	5371	12.63	–	–
Family-specific	409	5.35	66	0.47	512	4.25	604	1.66	367	0.86	8	0.25
Family-shared	39	0.51	1972	13.90	–	–	844	2.32	243	0.57	0	–
Order-specific	237	3.10	–	–	–	–	127	0.35	28	0.07	2	0.06
Order-shared	–	–	–	–	1941	16.10	51	0.14	3938	9.26	–	–
Class-specific	47	0.62	206	1.45	194	1.61	878	2.41	–	–	–	–
Class-shared	487	6.38	–	–	–	–	972	2.67	3951	9.29	–	–
Phylum-specific	5	0.07	–	–	542	4.50	15	0.04	–	–	–	–
Phylum-shared	219	2.87	1087	7.66	663	5.50	3143	8.64	3180	7.48	163	5.07
Domain-specific	26	0.34	–	–	–	–	–	–	–	–	–	–
Domain-shared	233	3.05	22	0.16	28	0.23	216	0.59	96	0.23	3	0.09

Note: The averages of species-specific, phylum-shared and domain-shared are 58.79%, 6.20% and 0.73%, respectively. Because Actinobacteria contains only one class, class-specific mini-proteins are equal to the phylum-specifics'; similarly, Spirochaetes contains one class and one order, so the order-specific mean the phylum-specific. The hypothetical proteins account for 82.80%, 86.51%, 85.77%, 79.91%, 84.49% and 95.37% of the species-specific in Euryarchaeota, Actinobacteria, Cyanobacteria, Firmicutes, Gammaproteobacteria and Spirochaetes, respectively.

From Table 2, it is clear that the species-specific mini-proteins are the majority in all of the phyla (average proportion is 58.79%), suggesting that these proteins potentially take on some unique functions that contribute to the adaptation of organisms to different habitats. However, 85.81% of them are annotated as “hypothetical protein” and the authenticity of their existence has not been confirmed. In contrast, shared or conserved proteins account for a small fraction, with 6.20% phylum-shared and 0.73% domain-shared (conserved in both Archaea and Bacteria). It is worthy of attention that Firmicutes comprise a larger proportion of these shared mini-proteins than any other bacterial phyla. In addition, although the proportion of hypothetical proteins is low among the conserved proteins, some hypothetical proteins are phylum-shared and domain-shared proteins. However, most phylum-shared proteins are well-characterized, such as, in the phylum-shared class, various ribosomal proteins, cold shock protein, translation initiation factor IF-1; in the domain-shared class rubredoxin, transcriptional regulator and gas vesicle protein (see Table 3 for a complete list).

**Table 3:** Phylum-shared and domain-shared mini-proteins in phyla.

	Euryarchaeota		Actinobacteria		Cyanobacteria		Firmicutes		Gamma proteobacteria		Spirochaetes	
Phylum-shared	sum	219	sum	1087	sum	663	sum	3143	sum	3180	sum	163
	hypothetical protein	39	hypothetical protein	190	hypothetical protein	216	hypothetical protein	541	hypothetical protein	602	hypothetical protein	33
	ribosomal protein	135	ribosomal protein	482	ribosomal protein	214	ribosomal protein	1470	ribosomal protein	1132	ribosomal protein	83
	enzyme or submit	26	enzyme or submit	91	enzyme or submit	52	phage protein	231	cold-shock protein	387	translation initiation	
	Other	19	cold-shock protein	82	redoxin	29	cold-shock protein	179	enzyme or submit	219	factor IF-1	10
			redoxin	58	acyl carrier protein	24	DNA-binding protein	110	redoxin	175	acyl carrier protein	7
			translation initiation		translation initiation		translation initiation		DNA-binding protein	144	carbon storage regulator	5
			factor IF-1	37	factor IF-1	22	factor IF-1	100	translation initiation		GroES chaperone	4
			10 KD chaperonin	22	S4-like RNA		enzyme or submit	90	factor IF-1	112	other	21
			other	125	binding protein	15	redoxin	63	acyl carrier protein	105		
					other	91	acyl carrier protein	45	other	304		
							sporulation protein S	38				
							other	276				
Domain-shared	sum	223	sum	22	sum	28	sum	216	sum	96	sum	3
	hypothetical protein	99	hypothetical protein	7	hypothetical protein	12	hypothetical protein	78	hypothetical protein	21	hypothetical protein	1
	enzyme or submit	34	redoxin	5	gas vesicle protein	8	transcriptional		rubredoxin	32	rubredoxin	2
	redoxin	28	transcriptional		rubredoxin	4	regulator	49	transcriptional			
	transcriptional		regulator	4	enzyme or submit	4	DNA-binding protein	32	regulator	15		
	regulator	14	YHS domain protein	3			redoxin	14	cation transport			
	gas vesicle protein	11	other	3			enzyme or submit	13	regulator	7		
	other	47					other	30	other	21		

### Conservation of hypothetical proteins

**Figure 2:**
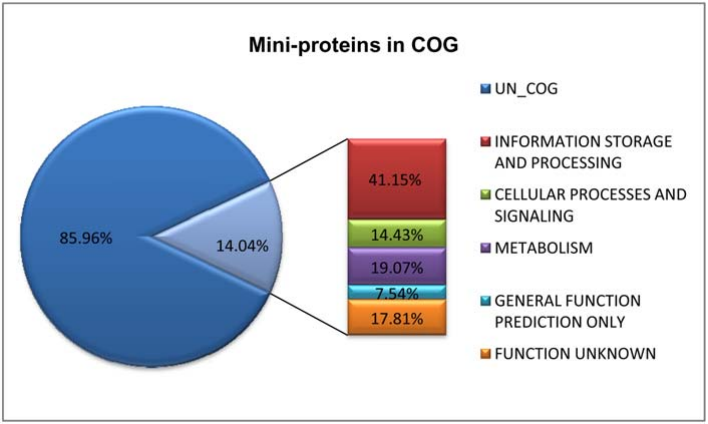
Mini-proteins in COG.

The aforementioned results show that hypothetical proteins accounted for a large proportion of mini-proteins, even among the conserved phylum-shared and domain-shared ones. In fact, about 70.03% or 126,670 mini-proteins are designated as hypothetical proteins, while merely 29.97% or 54,209 proteins possess functional or structural annotations. Moreover, 25,394 mini-proteins have been classified in the COG (Clusters of Orthologous Groups) database [11] and approximately 17.81% of them are unknown function (see Figure 2 for details). We further focused on these hypothetical proteins (also including uncharacterized protein and protein of unknown function, here together referred to as “hypothetical proteins”) to search for more conserved mini-proteins for better classification. We selected one strain from each genus that contains the most mini-proteins as representative in all phyla (Table 1) and analyzed these mini-proteins' conservation among all data (see Materials and Methods). We then picked out the mini-proteins whose homologous proteins are present in at least five of the phyla. As before, the five classes in Proteobacteria were also treated as distinct phyla.

As a result, we found many new groups of conserved mini-proteins and obtained 28 groups of proteins conforming to the above conditions. Then we compiled the data and searched for their functional domains on the Pfam [12] or InterProScan [13] websites (see Table 4 in details). These 28 groups of mini-proteins can be divided into three types. First, mini-proteins are well studied, with detailed functional and/or structural information, including group 01–07 and group 27–28. Second, they are the mini-proteins with domains named as DUF (Domain of Unknown Function) or UPF (Uncharacterized Protein Family). Third, the conservation is lower than those of above two types, whose domains are only found in Pfam-B, which supplements the databases' principal body (Pfam-A) and contains small families of proteins. The conservation of mini-proteins represented in Table 4 is generally high, with lowest similarity of 42% among these groups. Mini-proteins assembled in a group usually belong to the same domain, but proteins in groups 07, 18 and 20 include representatives from the two domains of Bacteria and Archaea. However, proteins of groups 18 and 20 are poorly characterized.

**Table 4:** Conservative analysis of hypothetical proteins in mini-proteins.

Serial Number	Domain Name	Blast Sum	Phylum	Positives	Identity	Annotation	Domain Description
group01	BMC	103	AcidobacteriaActinobacteriaCyanobacteriaFirmicutesFusobacteriaPlanctomycetesAlphaproteobacteriaBetaproteobacteriaDeltaproteobacteriaEpsilonproteobacteriaGammaproteobacteria	66.40%	7.40%	microcompartments protein;carboxysome shell protein;propanediol/ethanolamineutilization protein;	Bacterial microcompartments are primitive organelles composed entirely of protein subunits. The microcompartment is the carboxysome, a protein shell for sequestering carbon fixation reactions.
group02	PAAR-motif	16	AcidobacteriaBacteroidetesChloroflexiCyanobacteriaPlanctomycetesAlphaproteobacteriaDeltaproteobacteriaEpsilonproteobacteriaGammaproteobacteria	86.40%	24.30%	PAAR repeat-containing protein ;hypothetical protein ;	This motif is found usually in pairs in a family of bacterial membrane proteins. It is also found as a triplet of tandem repeats comprising the entire length in another family of hypothetical proteins.
group03	Plasmid-killer	20	ActinobacteriaCyanobacteriaAlphaproteobacteriaBetaproteobacteriaDeltaproteobacteriaGammaproteobacteria	74.20%	22.70%	plasmid maintenance system killer protein;hypothetical protein;	Several plasmids with proteic killer gene systems have been reported. All of them encode a stable toxin and an unstable antidote. The activation of those systems result in cell filamentation and cessation of viable cell production.
group04	Plasmid-Txe	40	AcidobacteriaActinobacteriaChloroflexiCyanobacteriaFirmicutesAlphaproteobacteriaBetaproteobacteriaDeltaproteobacteriaGammaproteobacteriaSpirochaetes	70.80%	5.70%	addiction module toxin (Txe/YoeB family);hypothetical protein;	The plasmid encoded Axe-Txe proteins act as an antitoxin-toxin pair.
group05	RHH-2	9	ActinobacteriaCyanobacteriaAlphaproteobacteriaBetaproteobacteriaGammaproteobacteria	74.20%	28.10%	putative transcriptional regulators(CopG/Arc/MetJ family);hypothetical protein;	This family of proteins is about 80 amino acids in length and their function is unknown. The proteins contain a conserved GRY motif. This family appears to be related to ribbon-helix-helix DNA-binding proteins.
group06	YcfA-like	11	FirmicutesAlphaproteobacteriaBetaproteobacteriaGammaproteobacteriaSpirochaetes	60.70%	17.90%	YcfA-like protein;hypothetical protein;	This family is similar to the YcfA protein expressed by E. coli. Most of these proteins are hypothetical proteins of unknown function.
group07	zf-UBP	12	AcidobacteriaActinobacteriaCyanobacteriaEuryarchaeotaDeltaproteobacteriaGammaproteobacteria	71.20%	26.00%	putative Zn-finger domain;hypothetical protein;	Zn-finger in ubiquitin-hydrolases and other protein
group08	DUF37	144	AcidobacteriaActinobacteriaBacteroidetesChlorobiChlamydiaeCyanobacteriaFirmicutesFusobacteriaAlphaproteobacteriaBetaproteobacteriaDeltaproteobacteriaGammaproteobacteriaSpirochaetesThermiThermotogae	46.50%	7.10%	alpha-hemolysin;protein of unknown function DUF37;hypothetical protein;	This domain is found in short (75 amino acid) hypothetical proteins from various bacteria. The domain contains three conserved cysteine residues.
group09	DUF196	13	ChlorobiFirmicutesAlphaproteobacteriaDeltaproteobacteriaGammaproteobacteria	86.60%	13.40%	CRISPR-associated protein;protein of unknown function DUF196;hypothetical protein;	This domain describes proteins of unknown function.Trm112p-like protein; The bacterial members are about 60–70 amino acids in length and the eukaryotic examples are about 120 amino acids in length. The C terminus contains the strongest conservation.
group10	DUF343	132	ActinobacteriaAlphaproteobacteriaBetaproteobacteriaDeltaproteobacteria	49.50%	5.30%	tetraacyldisaccharide -1-P 4-kinase ;protein of unknown function DUF343;hypothetical protein;	
group11	DUF370	46	FirmicutesCyanobacteriaThermotogaeChloroflexiDeltaproteobacteria	81.40%	20.60%	protein of unknown function DUF370 ;hypothetical protein;	Domain of unknown function
group12	DUF427	17	ActinobacteriaBacteroidetesChloroflexiCyanobacteriaThermiBetaproteobacteriaGammaproteobacteria	85.40%	27.10%	protein of unknown function DUF427 ;hypothetical protein ;	Domain of unknown function
group13	DUF433	6	AcidobacteriaChlorobiChloroflexiCyanobacteriaAlphaproteobacteria	70.20%	19.00%	protein of unknown function DUF433 ;hypothetical protein ;	Domain of unknown function
group14	DUF528	43	AcidobacteriaCyanobacteriaAlphaproteobacteriaBetaproteobacteriaDeltaproteobacteriaGammaproteobacteria	75.30%	10.40%	accessory protein involved in assembly of Fe-S clusters;protein of unknown function DUF528;hypothetical protein;	Domain of unknown function
group15	DUF891	11	CyanobacteriaAlphaproteobacteriaBetaproteobacteriaDeltaproteobacteriaGammaproteobacteria	65.50%	13.60%	protein of unknown function DUF891;hypothetical protein;	This family consists of hypothetical bacterial proteins of unknown function as well as phage Gp49 proteins.
group16	DUF1328	78	AcidobacteriaBacteroidetesAlphaproteobacteriaBetaproteobacteriaDeltaproteobacteriaGammaproteobacteria	57.00%	7.00%	putative inner membrane protein;hypothetical protein ;	This family consists of several hypothetical bacterial proteins of around 50 residues in length. The function of this family is unknown.
group17	DUF1458	37	ActinobacteriaChlorobiAlphaproteobacteriaBetaproteobacteriaGammaproteobacteria	56.10%	5.60%	protein of unknown function DUF1458 ;hypothetical protein ;	Members of this family are typically of around 70 residues in length. The function of this family is unknown.
group18	UPF0150	14	AcidobacteriaChloroflexiCyanobacteriaEuryarchaeotaFirmicutesDeltaproteobacteria	70.70%	6.10%	protein of unknown function UPF0150;hypothetical protein;	This domain is found next to a DNA binding helix-turn-helix domain, which suggests that this is some kind of ligand binding domain.
group19	pfam-B_8409	31	ChlorobiAlphaproteobacteriaBetaproteobacteriaEpsilonproteobacteriaGammaproteobacteriaThermi	73.80%	17.80%	predicted membrane protein;hypothetical protein;	
group20	pfam-B_11213	27	BacteroidetesEuryarchaeotaFirmicutesDeltaproteobacteriaEpsilonproteobacteriaGammaproteobacteriaThermi	63.50%	22.40%	hypothetical protein	
group21	pfam-B_20813	27	BacteroidetesCyanobacteriaPlanctomycetesBetaproteobacteriaGammaproteobacteria	50.00%	13.40%	hypothetical protein	
group22	pfam-B_20885	15	ActinobacteriaFirmicutesAlphaproteobacteriaBetaproteobacteriaGammaproteobacteria	44.60%	11.90%	uncharacterized conserved small protein like protein;hypothetical protein;	
group23	pfam-B_49955	22	BacteroidetesAlphaproteobacteriaBetaproteobacteriaEpsilonproteobacteriaGammaproteobacteria	64.30%	27.10%	oxygen-sensitive ribonucleoside-triphosphate reductase;hypothetical protein ;	
group24	pfam-B_108629	5	BacteroidetesAcidobacteriaAlphaproteobacteriaBetaproteobacteriaDeltaproteobacteria	47.40%	4.20%	hypothetical protein;	
group25	pfam-B_139336	7	ChlorobiCyanobacteriaAlphaproteobacteriaDeltaproteobacteriaGammaproteobacteria	75.30%	15.70%	hypothetical protein;	
group26	pfam-B_6607;pfam-B_9422[^1^]	35	BacteroidetesChlorobiAlphaproteobacteriaBetaproteobacteriaDeltaproteobacteriaEpsilonproteobacteriaGammaproteobacteria	51.80%	15.50%	hypothetical protein;	
group27	signal-peptide;transmembrane-regions	43	AcidobacteriaAlphaproteobacteriaBetaproteobacteriaDeltaproteobacteriaGammaproteobacteria	55.10%	10.10%	conserved hypothetical membrane protein;hypothetical protein;	
group28	TRASH;zf-HIT[^2^]	31	CyanobacteriaAlphaproteobacteriaBetaproteobacteriaGammaproteobacteriaThermi	42.00%	12.50%	zinc finger protein;hypothetical protein;	TRASH :metallochaperone-like domainzf-HIT :This presumed zinc finger contains up to 6 cysteine residues that could coordinate zinc.

Note: [1] Pfam-B_6607 and pfam-B_9422 are continuous; [2] Different domains are searched through different sequences.

### Evolutionary analysis of domains

**Figure 3:**
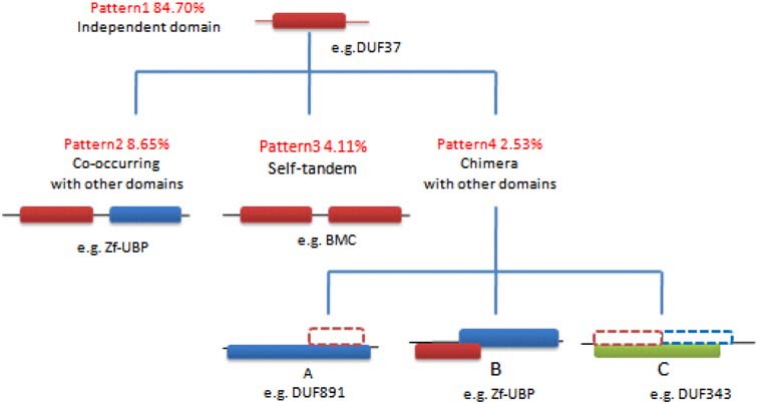
Patterns of domains. Dashed lines mean the domain have been evolved to a part of other domain or protein family's conserved region.

We further investigated the domains (or motifs and conserved regions) within the conserved hypothetical proteins in Table 4 as well as the phylum-shared and the domain-shared proteins in Table 3, and observed four patterns in the process of their evolution (see in Figure 3). We noticed that (i) these domains are highly conserved and widespread. Four domains, Plasmid-killer, Plasmid-Txe, RHH-2 and DUF370, were specific to Bacteria; other domains were conserved in Bacteria as well as in Viruses, Archaea and Eukarya. Except for Zf-UBP, which is mainly represented in eukaryotes, all other domains mainly exist in Bacteria. This suggests that the domains in mini-proteins are more likely to contribute to the bacterial species rather than that of eukaryotes; (ii) these domains seem to have evolved independently in mini-proteins, except for the PAAR-motif, which is often observed in tandem repeats. However, with the extension of protein lengths, the domains developed at least two patterns, except for those with independent evolution such as RHH-2, DUF37, DUF196, DUF370 and DUF528; (iii) independent domains seem to be more frequent than any of the three patterns, whereas self-tandem is the major pattern for PAAR-motifs and, chimera with other domains is the major pattern for Zf-UBP and YHS domains.

Domains represent the functional and evolutionary units of proteins, and almost all mini-proteins contain one domain. Results of our analysis indicate that individual domains evolve independently. Most domains develop new patterns during long-term evolution although the patterns of independent domains account for the majority in terms of number. In the course of evolution, proteins have a general tendency to fuse into two or multi-domain units from the single unit, which may help proteins develop new functions. As shown in Figure 3, proteins in pattern 2 achieve the functional integration through combination with different domains, which is a predominant route of protein evolution. In regard to pattern 3, it is also a relatively common method of protein evolution from single to multiple domains. The number of self-tandem domains is variable in proteins. For example, BMC (bacterial microcompartment) is always tandem with two repeats, but in proteins CSD (cold-shock domain) is not stabilized and tandem up to six domains.

A typical example of independent evolution is DUF37, which originates from group 08 in Table 4. This group of mini-proteins includes the largest searched sequences and covers all phyla of Bacteria, 144 total sequences of the 15 phyla. The majority of them are hypothetical proteins or proteins of unknown function, except 4 proteins that are annotated alpha-hemolysin, which is a bacterial toxin that can assemble a transmembrane pore. In the InterPro database [14], we detected 653 proteins possessing this domain, including one sequence in virus, 9 sequences in green plants and 643 sequences in Bacteria. Also, these proteins do not comprise another domains any more, which suggests that DUF37 evolved independently.

**Figure 4:**
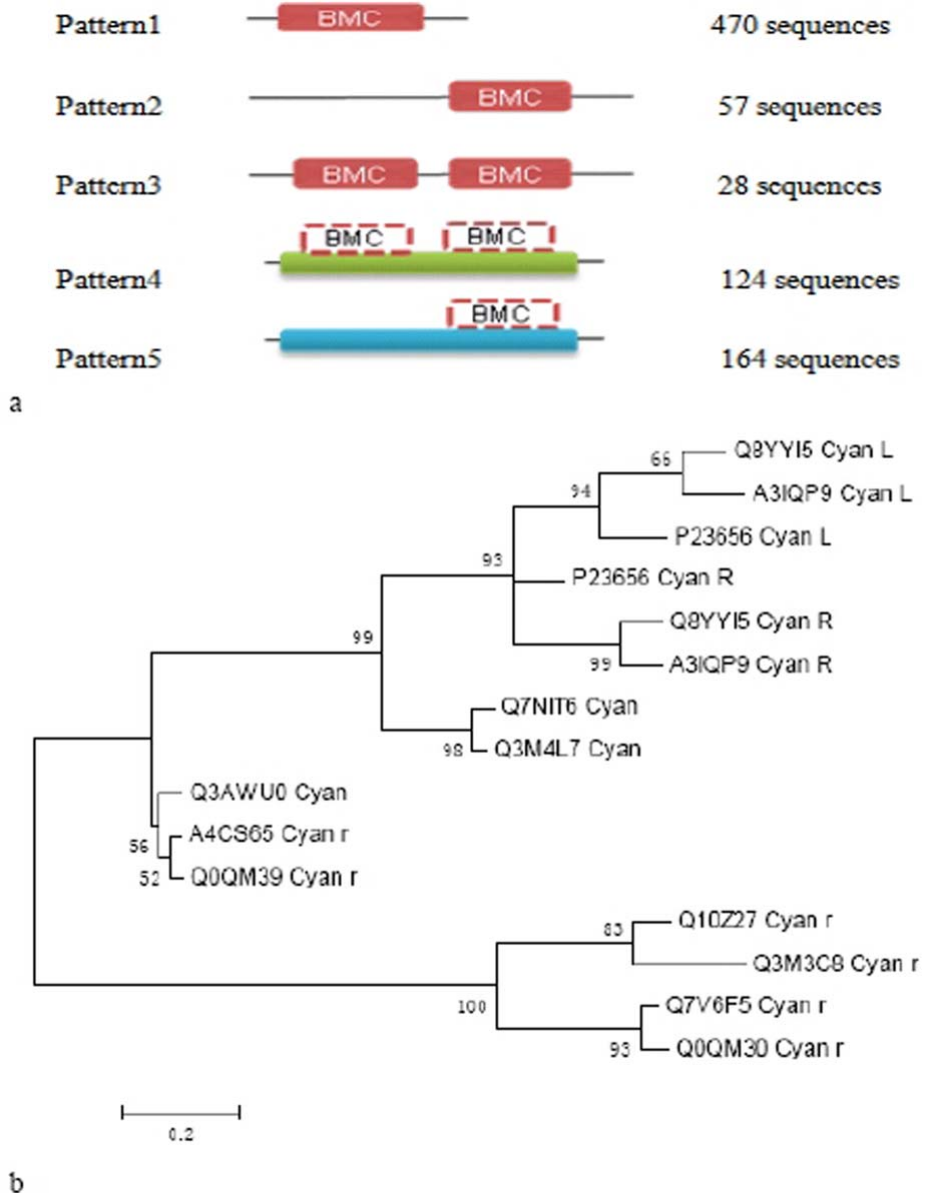
A: Patterns of BMC domain. Dashed lines mean the domain have been evolved to a part of other domain or protein family's conserved region. Green represents IPR011238 (polyhedral organelle shell protein PduT) and IPR009193 (polyhedral organelle shell protein, EutL/PduB type) in pattern4; blue represents IPR009193 (polyhedral organelle shell protein, EutL/PduB type) and IPR009307 (ethanolamine utilization EutS) in pattern 5. They are all polyhedral organelle shell proteins. B: Phylogeny of BMC domain in Cyanobacteria. Letter L and R represent the left and right domain in pattern 3 or 4, respectively. Letter r represents the domains in pattern 2 or 5.

In addition, many domains consist of at least two patterns. A good example is BMC within the group 01 of mini-proteins which involves 103 sequences of 11 phyla. We found that 843 proteins contain this domain in the InterPro database and summarized its evolutionary patterns. From Figure 4A, we can find that beside independent domain (62.51%), BMC has developed other two patterns: self-tandem (18.04%) as well as chimera with other domain (19.45%). In spite of different patterns, the proteins still possess similar functions, which indicate that one BMC domain is necessary to exert its function instead of requiring tandem of two BMC domains. We further investigated its phylogeny and used Cyanobacteria as an example (Figure 4B). It is clearly observed that the self-tandem and chimera with other domain pattern are divergent from independent domain because the BMC domains in pattern 3 or 4 and pattern 2 or 5 form two independent clusters, respectively. The left and right domains are clustered in pattern 3 or 4, respectively. This implies that the existence of tandem domains may not be the result of the domain duplication, rather the transfer of the domains between proteins.

## Discussion

Our study collected all annotated mini-protein sequences from the sequenced genomic data and carried out the comprehensive and systemic analysis, although previously there were a few sporadic reports about the structural and functional analyses of mini-proteins [2]–[8]. We found that the number of mini-proteins gradually increases with their length in amino acids. In particular, mini-proteins in the range of 70AA<length≤100AA account for 57.79% of the total, which concurs with the view that the size of a protein domain is generally below 100 amino acids [15]. This is the reason why we have chosen this length as the cut-off of proteins for analysis. With regard to smaller proteins, it has been suggested that mini-proteins (40–50AA) can exhibit a well defined three-dimensional structure through disulfide bridges, metal ion binding and specific hydrophobic interactions [4]. However, *Samuel et al.* reported that mini-proteins with just 20 amino acids can also adopt well-defined globular shapes [16]. Surprisingly, our analysis indicated that the number of mini-proteins ≤30AA is very low. It is possible that many small mini-proteins may have been filtered out during annotation, grossly under estimating the actual number of mini-proteins.

Our results indicate that mini-proteins are numerous, accounting for an average of 10.99% of all genomic data in Bacteria and Archaea. Despite the enormous total sum, distribution of the mini-proteins exhibits remarkable variation among different strains. For example, more than 30% of proteins encoded by the genome are mini-proteins in two strains of *Prochlorococcus marinus* (30.83% and 30.30%) as well as *Anaplasma phagocytophilum HZ* (33.39%). *A. phagocytophilum HZ* represents the greatest percentage of mini-proteins encoded on the genome. By contrast, *Clostridium tetani* in Firmicutes, represents a unique strain with no known mini-proteins encoded on its genome. Interestingly, both the maximum and the minimum belong to the bacterial domain. Consequently, the range of variation of mini-protein content in Bacteria (0.16%–33.39%) spans much greater than in archaea (7.83–18.23%). Although the concrete biological significance is unknown, we speculate that this phenomenon may relate to the fact that ecological conditions of bacterial species are more diverse and complicated than that of archaeal species which are mostly in constant but extreme environments [17], [18].

In addition, even among closely related species, the relative proportions of the mini-proteins vary greatly. In *Clostridium*, except for *C. tetani* which encodes no mini-proteins, the other nine strains all encode mini-proteins, ranging from 8.27% to 14.25% of the total number of proteins. Species of this genus are ubiquitous in soils, aquatic sediments and the intestinal tracts of animals and humans; hence they display metabolic and biological diversity. Surprisingly, ferredoxin, ATP synthase subunit C and 50S ribosomal protein L27 are less than 100 amino acids and belong to mini-proteins in the nine strains, but in *C. tetani*, they are 290AA, 333AA and 101AA long, respectively. It is plausible that even subtle changes in the environment may become a selective pressure for mini-proteins, and the differences among *Clostridium* are the result of multifactor influence. However, it is difficult to determine which environmental factors affect the evolution of mini-proteins. Nonetheless, a few examples can provide some clues to certain extent. For instance, the aforementioned *Anaplasma* includes two sequenced species, *A. phagocytophilum HZ* (contains 33.39% mini-proteins) and *A. marginale str. St. Maries* (contains 7.59% mini-proteins). They are both obligate intracellular pathogens, but they inhabit granulocytes and erythrocyte, respectively [19], [20], therefore, differences in the host intracellular environments might account for the significant differences in relative proportion of the mini-proteins. Moreover, the proportions of mini-proteins in the genome are actually dissimilar between different isolates of the same species, such as two strains in Spirochaetes, *Leptospira interrogans serovar Copenhageni str. Fiocruz L1-130* and *L. interrogans serovar Lai str. 56601*, contain 10.74% and 28.71% mini-proteins, respectively. We speculate that this difference between mini-protein proportions may reflect different selection pressures the two strains are exposed to, resulting in different leptospiral serovars that are derived from structural heterogeneity in the carbohydrate component of lipopolysaccharides [21].

Our results reveal that one characteristic of mini-protein data is that species-specific proteins predominate, whereas conserved proteins are the minority, which ought to be the chief reason for the fluctuations in mini-protein content. Why are species-specific proteins so numerous? We speculate several possible reasons: first, the mini-proteins help organisms to adapt to the diverse and distinctive ecological niches, thus many of them are species-specific. Particularly in Bacteria, some species freely live in various aqueous or terrestrial environments, while others are intracellular parasites, obligate and facultative parasites of animals and plants. Second, some of the mini-proteins are short remnants of longer genes that were present in their early ancestors. Third, some proteins probably evolved too rapidly to maintain homologues and intermediate sequences. Fourth, similar proteins have been incorrectly annotated [22]. In fact, mini-proteins are capable of being very good candidates for the species-specific. On one hand, the vast majority of mini-proteins contain one domain which lets them exert functions simply and directly through protein-protein interactions or binding DNA or RNA sequences. On the other hand, since mini-proteins require less to translate and fold, organisms use them to regulate relevant pathways and respond to subtle changes in the environment, which accords with the hypothesis that organisms tend to minimize costs of protein biosynthesis [23]. Additionally, the amount of conserved proteins is less, but most of them are necessary for the survival of organisms, especially those phylum-shared and domain-shared ones.

Another characteristic of mini-protein data is that although hypothetical proteins are the majority and the proteins with known functions are the minority, the functions of mini-proteins are diverse. As shown in Figure 2, mini-proteins are involved in broad functional classes, including information storage and processing, cellular processes and signalling, and metabolism. In fact, they are distributed in nearly all subclasses of three larger classes, except for RNA processing and modification, nuclear structure, cytoskeleton and extracellular structures (data not shown). This result implies that regulatory and metabolic proteins are more common than constitutive or structural proteins, which can also be observed clearly from phylum-shared and domain-shared proteins. As previously mentioned, some of 299 mini-proteins in the *S. cerevisiae* genome are required for growth under genotoxic conditions including exposure to hydroxyurea (HU), bleomycin and ultraviolet (UV), suggesting that they play important roles to harsh environmental conditions [2]. Furthermore, the proportion of hypothetical proteins is very high, about 70.03%. This might be due to the fact that (i) a great deal of mini-proteins are species-specific; (ii) some of the mini-proteins might be incorrectly annotated; and (iii) there are technical difficulties in identifying the functions of mini-proteins. However, we discovered that even in hypothetical proteins there are still a fraction of conserved sequences, including conserved proteins at each taxonomic level and 28 groups of proteins spanning beyond five phyla. These will be useful for us to correctly annotate proteins and further explore the function and evolution of mini-proteins; especially those highly conserved sequences listed in Table 4 which are more biologically significant and will be an emphasis of our future studies.

Mini-proteins have received significantly less attention from researchers due to the constraints of experimental and bioinformatic approaches. Here, we investigated the annotated mini-proteins from the sequenced genomic data and discovered some overall rules which could establish a foundation for further studies. However, the answers to many questions remain elusive and wait to be resolved in the future. They include (i) how to identify potentially more mini-proteins in various genomes; (ii) how to confirm the functions of identified mini-proteins; (iii) what are the biological functions of the mini-proteins; and (iv) what are the driving forces for the evolution of the mini-proteins.

## Materials and Methods

We collected 532 completed prokaryotes genomes from the National Center for Biotechnology Information (NCBI) up to date the 2nd July, 2007; and extracted all annotated protein sequences ≤100 amino acids in their chromosomes and plasmids, as the length of one domain is usually below that cut-off value. Moreover, every strain was classified according to the NCBI taxonomy database.

We first analyzed the overall length distribution of mini-proteins and described the main characteristics of the each phylum. And then, to detect the special or shared mini-proteins of six representative phyla we started by carrying out a BLASTP search of every mini-protein sequence in one phylum against all mini-protein data we extracted. In regard to the last results, we recorded the matches for each protein sequence with an E-value lower than 10^−5^, sequence identity higher than 60% and filtered low-complexity sequences.

In addition, to explore the conservation of mini-proteins in all phyla we also carried out BLASTP searches using mini-proteins queries from a representative species for every genus against all mini-protein data with parameters as previously described. Amino acid sequence alignments were obtained with Clustalx software [24]. For the domain analysis, we investigated them using the Pfam or InterPro websites and proteins' sequences include all prokaryotic and eukaryotic data. Moreover, we used Mega [25] software (bootstrapped neighbor-joining method) for phylogenetic reconstructions.
